# Analysis of Clinician and Patient Factors and Completion of Telemedicine Appointments Using Video

**DOI:** 10.1001/jamanetworkopen.2021.32917

**Published:** 2021-11-04

**Authors:** Bradley H. Crotty, Noorie Hyun, Alexandra Polovneff, Yilu Dong, Michael C. Decker, Natalie Mortensen, Jeana M. Holt, Aaron N. Winn, Purushottam W. Laud, Melek M. Somai

**Affiliations:** 1 Collaborative for Healthcare Delivery Science, Center for Advancing Population Science, Medical College of Wisconsin, Milwaukee; 2 Inception Labs at Froedtert & Medical College of Wisconsin Health Network, Milwaukee; 3 Department of Emergency Medicine, Medical College of Wisconsin, Milwaukee; 4 School of Nursing, University of Wisconsin, Milwaukee

## Abstract

**Importance:**

Telemedicine provides patients access to episodic and longitudinal care. Policy discussions surrounding future support for telemedicine require an understanding of factors associated with successful video visits.

**Objective:**

To assess patient and clinician factors associated with successful and with failed video visits.

**Design, Setting, and Participants:**

This was a quality improvement study of 137 846 scheduled video visits at a single academic health system in southeastern Wisconsin between March 1 and December 31, 2020, supplemented with patient experience survey data. Patient information was gathered using demographic information abstracted from the electronic health record and linked with block-level socioeconomic data from the US Census Bureau. Data on perceived clinician experience with technology was obtained using the survey.

**Main Outcomes and Measures:**

The primary outcome of interest was the successful completion of a scheduled video visit or the conversion of the video visit to a telephone-based service. Visit types and administrative data were used to categorize visits. Mixed-effects modeling with pseudo *R*^2^ values was performed to compare the relative associations of patient and clinician factors with video visit failures.

**Results:**

In total, 75 947 patients and 1155 clinicians participated in 137 846 scheduled video encounters, 17 190 patients (23%) were 65 years or older, and 61 223 (81%) patients were of White race and ethnicity. Of the scheduled video encounters, 123 473 (90%) were successful, and 14 373 (10%) were converted to telephone services. A total of 16 776 patients (22%) completed a patient experience survey. Lower clinician comfort with technology (odds ratio [OR], 0.15; 95% CI, 0.08-0.28), advanced patient age (66-80 years: OR, 0.28; 95% CI, 0.26-0.30), lower patient socioeconomic status (including low high-speed internet availability) (OR, 0.85; 95% CI, 0.77-0.92), and patient racial and ethnic minority group status (Black or African American: OR, 0.75; 95% CI, 0.69-0.81) were associated with conversion to telephone visits. Patient characteristics accounted for systematic components for success; marginal pseudo *R*^2^ values decreased from 23% (95% CI, 21.1%-26.1%) to 7.8% (95% CI, 6.3%-9.4%) with exclusion of patient factors.

**Conclusions and Relevance:**

As policy makers consider expanding telehealth coverage and hospital systems focus on investments, consideration of patient support, equity, and friction should guide decisions. In particular, this quality improvement study suggests that underserved patients may become disproportionately vulnerable by cuts in coverage for telephone-based services.

## Key Points

**Question:**

Which patient and clinician factors are associated with a successful or failed video visit?

**Findings:**

This quality improvement study of 137 846 video visits showed an overall 90% success rate. Patient rather than clinician factors were more systematically associated with successful completion of video visits, and clinician comfort with technology was associated with successful video visits or conversion to telephone visits.

**Meaning:**

The findings suggest that, as policy makers consider expanding telehealth coverage and hospital systems focus on investments, consideration of patient support, equity, and friction should be kept in the forefront.

## Teaser

This quality improvement study evaluates patient and clinician factors to assess which factors are associated with the successful completion or failure of telemedicine video appointments.

## Introduction

Although telemedicine has been practiced for decades, its necessity reached new heights during the COVID-19 pandemic. Prior to this pandemic, direct-to-consumer telemedicine use was driven by a relatively small segment of the health care consumer population.^1,2,3^ However, during the COVID-19 pandemic, the rapid expansion of telemedicine in 2020 was facilitated by legislative and executive changes during the public health emergency that removed the “originating site” provision and increased restriction of in-person visits. With shortages of personal protective equipment and uncertainty regarding viral transmission, particularly with high viral transmission levels in several regions in the United States, health care shifted to virtual visits at a quick pace.^4^ Health care organizations rapidly deployed and scaled up virtual visits in efforts to adjust access to care, which meant that a majority of patients were experiencing telemedicine and virtual health care for the first time.^5,6,7^

The rapid transition to virtual health care raised important questions concerning equity of access and the design of health care experiences^8^ and introduced an array of new technical challenges for stakeholders, including patients, clinicians, and health care systems. Telemedicine requires access to a smartphone or a computer, navigation of several screens to access the visit, and a stable internet connection that allows for fluent conversation and examination. Patients and physicians reported difficulties with platforms, accessing visits, and quality connections.^6^ These friction points are more likely to challenge less socioeconomically advantaged patients, which may contribute to health disparities.^9^ Understanding and exploring the difficulties that arose during the public health emergency is vital to address ongoing accessibility problems and provide adequate support for a sustainable medium of health care delivery in the future.

As governments, payers, and systems plan for telemedicine at the culmination of the public health emergency, an understanding of the limitations of telemedicine in reaching patients is critical to guide decisions and investments.^10,11^ Prior studies have reported that patients at a relative socioeconomic disadvantage are less likely to schedule video visits as opposed to audio-only visits.^12,13,14^ Using hierarchical regression analysis techniques, Rodriguez et al^12^ also identified that much of the variability in choosing the modality for telemedicine rested with clinicians rather than with patients. In the present study, we sought to identify factors that were associated with the inability to complete a video visit at both the patient and clinician levels to better understand where variability resided.

## Methods

### Design, Setting, and Participants

This quality improvement study was conducted between March 1 and December 31, 2020, at Froedtert & the Medical College of Wisconsin Health Network, an academic health system based in southeastern Wisconsin. All patients scheduled for a video-based visit during this period were included across primary and specialty care. The health system concurrently used 2 video visit platforms during this period. The first was integrated into the electronic health record (EHR) and patient portal. The second was a nonintegrated solution that delivered a link to patients through a text message to join the visit through a web-based video-conferencing platform. Visits that were not successful using the integrated solution could use the nonintegrated solution as a backup. Clinics generally chose a preferred option that was the default mode for all visits in that department. Reporting of this study followed the Standards for Quality Improvement Reporting Excellence (SQUIRE) reporting guideline.^15^ The institutional review board of the Medical College of Wisconsin approved the study and granted approval of a waiver of the Health Insurance Portability and Accountability Act authorization requirements at 45 CFR 164. No one received compensation or was offered any incentive for participating in this study.

### Outcomes

Video visits were considered a success if the visit service was completed. Video visits were considered a failure if they resulted in a change to a telephone-based service. Both EHR and billing data were used to determine the video visit outcome. For the purposes of this analysis, patient cancellations (other than to reschedule as a same-day telephone service) and no-show visits were excluded.

### Identification of Independent Variables

We obtained information about patients through the EHR, including age at visit, race and ethnicity, and their insurance or payer. Patient median income was estimated from the US Census Bureau.^16^ High-speed internet availability was determined through Federal Communications Commission data linked to block-level data.^17^ Clinician information was obtained from the clinician’s record within the EHR and included physician specialty and the number of prior successful video visits.

Patient experience data were available for a subset of patients who responded to after-visit surveys. The survey data included patient ratings of how well the visit met their needs, their perception of how comfortable the clinician was with technology, and information about the type of device used to access the visit.

### Statistical Analysis

Descriptive statistics were summarized to characterize patients, clinicians, and visits. The weekly number of appointments by platform and the video visit success rates (the number of successful video visits across the total number of visits during the week) were depicted to show the video visit success rates with time. We applied logistic, multiple mixed-effects regressions for visit outcome (success vs failure) to 2 samples, including only the first visit for each patient (analysis 1) and only visits from patients with more than 1 visit (analysis 2). We used a mixed-effects model to account for the correlated data among visits by the same clinician and patient. In the mixed-effects models, potential confounding factors associated with successful video visits included patient characteristics (eg, age, race and ethnicity, medical insurance, geocode-based median income, comorbidity, the device used for the visit, and the number of prior successful video visits) and clinician factors, including title and specialty, perceived comfort with technology (as assessed by patient survey), and the number of previously successful video visits. We also considered clinical location as a potential factor.

We first selected statistically significant factors from univariate analysis and developed the final model using composite criteria, including stepwise variable selection based on Akaike information criteria, residuals, and reliable standard error estimates.^18,19,20^ Bernstein polynomials and spline curves were used to approximate the log odds of video visit success for the number of prior video visit successes to allow for the detection of a nonlinear association with prior successful video visits without specifying cutoff points. The final model included random intercept effects for clinicians (analysis 1) and random intercept effects for clinicians and patients (analysis 2).

To address whether patient or clinician factors were relatively more strongly associated with the successful video visit, we compared the final model given in the eTable in the Supplement with reduced models, which included only patient factors and clinician factors from the final model produced in analysis 2, respectively, in terms of pseudo *R*^2^ values.^18,21^ The pseudo *R*^2^ value is the proportion of the variance explained by the fixed effects only (marginal) and mixed effects (conditional) over the total variance consisting of the 3 variance components of fixed effects, random effects, and error. The pseudo *R*^2^ values range from 0 to 1, similar to the ordinal *R*^2^ value. A decrease in the pseudo *R*^2^ value after removing patient or clinician factors indicates that those respective factors may be associated with the variation in successful video visits in the final model. We used 500 bootstrap resamples with replacement to calculate 95% CIs for the pseudo *R*^2^ value.

All analyses were conducted using R, version 4.0.3 (R Project for Statistical Computing). A 2-sided value of *P* < .05 was considered statistically significant.

## Results

In total, 75 947 unique patients were included in the analysis (Table 1). Of the included patients, 17 190 (23%) were 65 years or older, 10 272 (14%) were African American or Black, 233 (<1%) were Alaska Native or American Indian, 1540 (2%) were Asian, and 61 223 (81%) were White. A total of 29 588 patients (39%) were Medicare or Medicaid beneficiaries, and 43 357 patients (57%) were under managed care programs. Among all included patients, 16 776 (22%) with completed visits responded to a patient experience survey.

**Table 1.** Patient Demographic Characteristics Among Scheduled Video Visits

Characteristic	Experience survey, No. (%)^a^		Total No. (%)^a^
	Nonrespondents	Respondents	
No.	59 171	16 776	75 947
Age, y			
18-40	23 050 (39)	2777 (17)	25 827 (34)
41-65	25 228 (43)	7702 (46)	32 930 (43)
66-80	8933 (15)	5616 (33)	14 549 (19)
>80	1960 (3)	681 (4)	2641 (4)
Race and ethnicity			
African American or Black	9124 (15)	1148 (7)	10 272 (14)
Alaska Native or American Indian	185 (<1)	48 (<1)	233 (<1)
Asian	1304 (2)	236 (1)	1540 (2)
Native Hawaiian or Other Pacific Islander	58 (<1)	9 (<1)	67 (<1)
White	46 265 (78)	14 958 (89)	61 223 (81)
Other or unknown^b^	2235 (4)	377 (2)	2612 (3)
Insurance			
Managed care	34 836 (59)	8521 (51)	43 357 (57)
Commercial	1046 (2)	191 (1)	1237 (2)
Medicaid	7120 (12)	723 (4)	7843 (10)
Medicare	14 691 (25)	7054 (42)	21 745 (29)
Others	534 (2)	116 (<1)	650 (1)
Not available	944 (2)	171 (1)	1115 (2)
Median income, $			
9500-45 000	12 474 (21)	2203 (13)	14 677 (19)
45 001-75 000	24 595 (42)	7000 (42)	31 595 (42)
75 001-213 000	12 486 (21)	4464 (27)	16 950 (22)
Not available	9616 (16)	3109 (19)	12 725 (17)
High-speed internet availability^c^			
High	20 559 (35)	6745 (40)	27 304 (36)
Medium	7697 (13)	1616 (10)	9313 (12)
Low	21 709 (37)	5455 (33)	27 164 (36)
Not available	9206 (16)	2960 (18)	12 166 (16)
Device used for the visit			
Android phone or iPhone	NA	9058 (54)	9058 (12)
Laptop or computer	NA	2586 (15)	2586 (3)
Other smartphone	NA	989 (6)	989 (1)
Tablet	NA	1594 (10)	1594 (2)
Not available	59 171 (100)	2549 (15)	61 720 (81)

Abbreviation: NA, not applicable.

^a^ Percentages may not equal 100 because of rounding.

^b^ As categorized in the electronic health record.

^c^ Residential fixed high-speed connections derived from Federal Communications Commission data. High denotes more than 800 connections capable of download speeds of at least 10 megabits per second and upload speeds of 1 megabit per second per 1000 households; medium, 601 to 800 connections per 1000 households; and low, 600 or fewer connections per 1000 households.

Clinicians (n = 1155) represented a broad array of specialties, including 240 (21%) in primary care areas and 340 (29%) in internal medicine subspecialties (Table 2). As judged by their patients through survey responses, a majority of clinicians were assessed to have a high degree of comfort with technology (801 of 930 respondents [86%] strongly agreed).

**Table 2.** Clinician Characteristics

Characteristic	Clinicians, No. (%)^a^
Specialty	
No.	1155
Anesthesia and pain management	10 (<1)
Behavioral health	49 (4)
Dermatology	25 (2)
Gynecology	68 (6)
Internal medicine subspecialty	340 (29)
Neurology	76 (7)
Occupational health	25 (2)
Ocular services	12 (1)
Primary care	240 (21)
Radiology	29 (2)
Rehabilitation	15 (1)
Surgery	265 (23)
Perception of comfort with technology^b^	
No.	930
Strongly agree	801 (86)
Agree	99 (11)
Neutral	24 (3)
Disagree	3 (<1)
Strongly disagree	3 (<1)

^a^ Percentages may not equal 100 because of rounding.

^b^ Mode value of assessments by patients through surveys.

We analyzed 137 846 billable scheduled video visits from March through December 2020 (Table 3). Of them, 123 473 (90%) were successful, and 14 373 (10%) were converted to telephone services. The clusters of clinicians and patients were not nested but partially overlapped. The median cluster size of clinicians (the number of all types of visits per clinician) was 50 (IQR, 11-152). Nearly 60% of the visits were the patient’s only visit within the study period. Among patients with more than 1 visit, the median number of visits was 3 (IQR, 2-5). Video visits were split between both the EHR or portal integrated solution (77 073 visits; 62%) and the nonintegrated solution (46 400 visits; 38%) (Figure).

**Table 3.** Video Visit Characteristics

Characteristic	Cases, No. (%)^a^	
	Success (n = 123 473)	Failure (n = 14 373)
Visit type		
Integrated video visit platform (EHR virtual)	77 073 (62)	8375 (58)
Nonintegrated video visit platform (virtual visit)	46 400 (38)	5998 (42)
Clinician specialty		
Anesthesia and pain management	1188 (1)	113 (1)
Behavioral health	9159 (8)	750 (5)
Dermatology	2035 (2)	137 (1)
Gynecology	4626 (4)	404 (3)
Internal medicine subspecialty	31 139 (25)	4818 (34)
Neurology	5773 (5)	789 (6)
Occupational health	573 (<1)	48 (<1)
Ocular services	41 (<1)	7 (<1)
Primary care	48 721 (40)	5224 (37)
Radiology	477 (<1)	129 (1)
Rehabilitation	931 (1)	168 (1)
Surgery	17 848 (15)	1704 (12)
Unknown	962	82
**Recommend to others** ^b^		
Responded	5323 (4)	501 (3)
Definitely, no	82 (2)	26 (5)
Probably		
No	210 (4)	36 (7)
Yes	937 (18)	125 (25)
Definitely, yes	4094 (77)	314 (63)
Did not respond or not available	118 150 (96)	13 872 (97)
**Addressed medical concern** ^b^		
Responded	15 733 (13)	1201 (8)
Strongly agree	12 545 (80)	844 (70)
Agree	2607 (17)	268 (22)
Disagree	91 (1)	15 (1)
Neutral	405 (3)	63 (5)
Strongly disagree	85 (<1)	11 (1)
Did not respond or not available	107 740 (87)	13 172 (92)

Abbreviation: EHR, electronic health record.

^a^ Percentages may not equal 100 because of rounding.

^b^ As assessed by patient survey responses (n = 16 776) where available.

**Figure.**
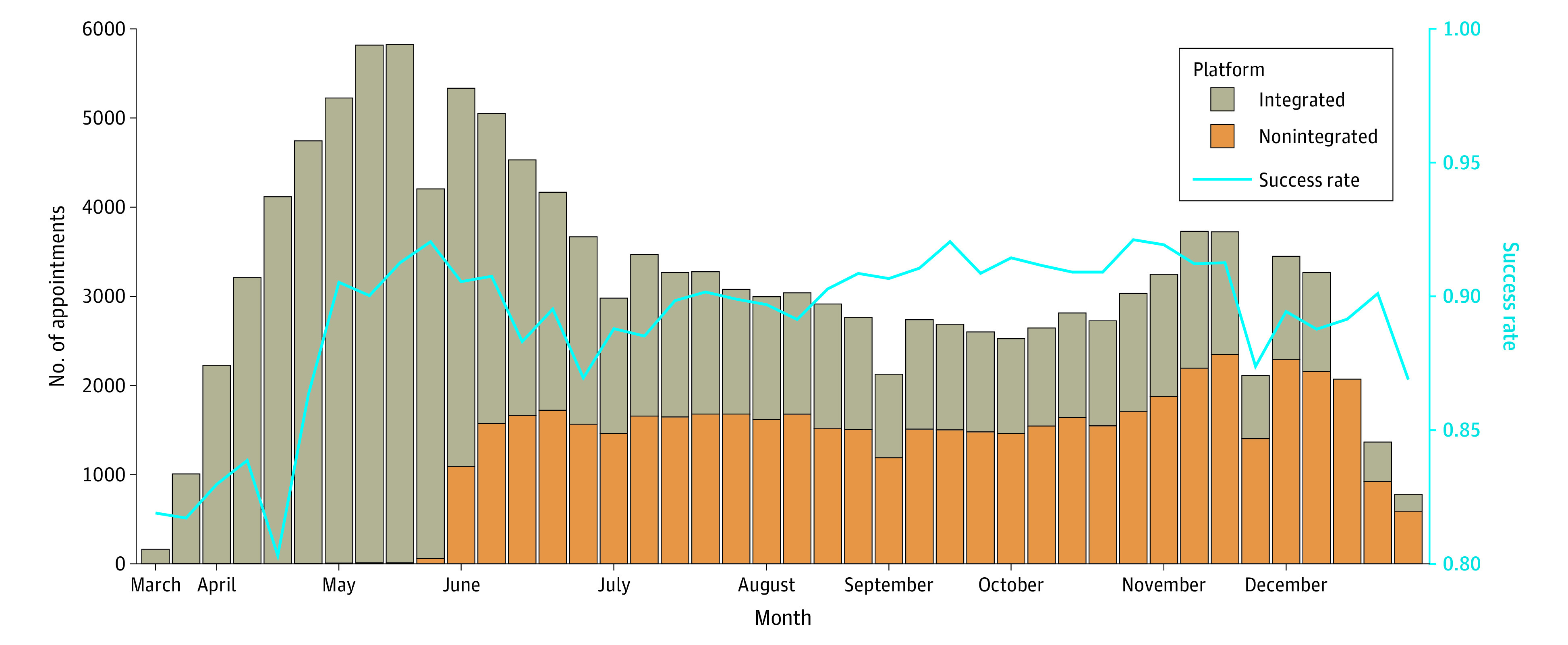
Video Visit Trends (by Platform) and Success Rate of Completing the Visit as a Video-Based Encounter

Because analyses 1 and 2 yielded similar results, we reported analysis 1 (Table 4). Conditional on scheduling a video visit, multivariate modeling indicated that patient income ($75 001-$213 000: odds ratio [OR], 1.18; 95% CI, 1.06-1.30) and patient use of a tablet or a laptop (OR, 1.41; 95% CI, 1.14-1.74) were associated with successful visits, whereas lower clinician comfort with technology (OR, 0.15; 95% CI, 0.08-0.28), advanced patient age (66-80 years: OR, 0.28; 95% CI, 0.26-0.30), racial and ethnic minority status of patients (Black or African American: OR, 0.75; 95% CI, 0.69-0.81), and using the nonintegrated video visit solution (OR, 0.57; 95% CI, 0.53-0.61) were associated with conversion to telephone visits (Table 4). We found that the learning curves in log odds for a successful video visit were similar for both patients and clinicians (eFigures 1 and 2 in the Supplement).

**Table 4.** Multicovariate Mixed-Effects Adjusted Odds Ratios for Having a Successful Video Visit

Characteristic	Odds ratio (95% CI)	*P* value	Overall *P* value
Clinician type			
Physician	1 [Reference]		
Advanced practice clinician	0.90 (0.77-1.06)	.20	
Clinician comfort with technology^a^			
Strongly agree	1 [Reference]		<.001
Agree	0.69 (0.58-0.83)	<.001	
Neutral	0.21 (0.16-0.26)	<.001	
Disagree	0.15 (0.11-0.22)	<.001	
Strongly disagree	0.15 (0.08-0.28)	<.001	
Not available	0.40 (0.29-0.56)	<.001	
Patient age, y			
18-40	1 [Reference]		<.001
41-65	0.55 (0.51-0.58)	<.001	
66-80	0.28 (0.26-0.30)	<.001	
>80	0.22 (0.20-0.25)	<.001	
Patient race and ethnicity			
African American or Black	0.75 (0.69-0.81)	<.001	<.001
Alaska Native or American Indian	0.64 (0.43-0.96)	.03	
Asian	0.97 (0.81-1.18)	.80	
Native Hawaiian or Other Pacific Islander	0.67 (0.30-1.50)	.30	
White	1 [Reference]		
Other or unknown^b^	0.86 (0.75-0.99)	.03	
Patient median income, $			
9500-45 000	1 [Reference]		.004
45 001-75 000	1.10 (1.02-1.19)	.01	
75 001-213 000	1.18 (1.06-1.30)	<.001	
Not available	1.19 (0.98-1.44)	.08	
Patient high-speed internet availability^c^			
High	1 [Reference]		.002
Medium	0.92 (0.86-0.98)	.01	
Low	0.85 (0.77-0.92)	<.001	
Not available	0.92 (0.76-1.11)	.40	
Patient device used for the visit			
Android or iPhone	1 [Reference]		<.001
Laptop or computer	1.41 (1.14-1.74)	.002	
Other smartphone	0.51 (0.41-0.63)	<.001	
Tablet	1.95 (1.49-2.55)	<.001	
Not available	0.68 (0.49-0.94)	.02	
Visit type			
Integrated video visit platform	1 [Reference]		
Nonintegrated video visit platform	0.57 (0.53-0.61	<.001	

^a^ As assessed by patient survey responses (n = 16 776) where available.

^b^ As categorized in the electronic health record.

^c^ Residential fixed high-speed connections derived from Federal Communications Commission data. High denotes more than 800 connections capable of download speeds of at least 10 megabits per second and upload speeds of 1 megabit per second per 1000 households; medium, 601 to 800 connections per 1000 households; and low, 600 or fewer connections per 1000 households.

We compared the final model with reduced models to determine whether patient or clinician factors were most strongly associated with the findings. The marginal pseudo *R*^2^ values were 23.3% (95% CI, 21.1%-26.1%), and the conditional pseudo *R*^2^ values were 42.3% (95% CI, 40.0%-44.5%). Those values decreased to 7.8% (95% CI, 6.3%-9.4%) for the marginal pseudo *R*^2^ values and to 29.4% (95% CI, 27.3%-31.3%) for the conditional pseudo *R*^2^ values when excluding patient factors in the model. Whereas the marginal *R*^2^ value did not change (*R*^2^ = 22.6%; 95% CI, 20.4%-25.4%), the conditional *R*^2^ value decreased to 26.4% (95% CI, 24.0%-29.0%) when excluding clinician factors. That elimination of patient factors significantly decreased the marginal pseudo *R*^2^ value, indicating that the patient factors were dominantly and systematically associated with video visit outcomes. Although the elimination of clinician factors decreased the conditional pseudo *R*^2^ value compared with the elimination of patient factors, it was not statistically significant.

## Discussion

Examining the roles of demographic characteristics, patient and clinician associated factors, and technology learning curves provides insight into the successes and failures of telemedicine. Clinicians were associated with some variability as a part of the equation, especially those working remotely, with poor network or with Wi-Fi network dropped connections, or those learning how to manage new equipment and workflows. However, this study showed that most of the variability in successful or failed video visits was associated with patient characteristics vs clinician characteristics, particularly regarding sociodemographic characteristics and age.

Sociodemographic characteristics, including internet connectivity, technology literacy, educational level, and technology support, are critical to the success of a video visit. Internet connection with sufficient bandwidth to facilitate a video visit is often a hurdle for various populations.^22^ One-fourth of rural households do not have access to broadband internet; the digital divide is also present in urban communities, emphasizing the necessity of more inclusive internet access.^9^ Video communication yields higher patient understanding and satisfaction compared with only telephone communication.^23^

In addition to differences by sociodemographic status, we observed differences by age, with patients older than 65 years being more likely to convert to a telephone visit. Older individuals may face more technology barriers, may have visual or movement disorders that make computing more difficult (especially on smaller devices), or may simply be more casual users of the internet.^24^ Despite those assumptions, individuals who are older likely have a higher need for virtual care associated with transportation challenges to and from appointments or other impairments or chronic ailments that make leaving the house difficult.^25^ In addition, telemedicine services may enable family members or caregivers, particularly those at a distance, to meaningfully participate in visits and to be a part of health care decision-making.

As patients and clinicians in the study population became more comfortable with technology, distinct learning curves were found in both user categories. The existence of a learning curve suggests that there are modifiable telemedicine program components, such as technical support or training, that may reduce video visit failures. Previous studies have shown that effective clinician training in telemedicine increases clinician confidence not only in using medical technology but in educating patients in how to have a successful video visit.^26^ The patients’ learning curve was notably longer to achieve a successful video visit. This outcome may result from the irregular cadence of virtual care visits that does not allow for persistent learning or repetition, whereas clinicians perform multiple video visits on a single day. Future patient and clinician education on best virtual visit practices and patient accessibility to the internet or technology may help smooth experiences and shorten learning curves.

With the rapid implementation of virtual telemedicine during the COVID-19 pandemic, it can be safely assumed that several traditionally in-person medical encounters will be virtual for the foreseeable future.^27^ As such, examining factors that are associated with the successes and failures of video visits provides insights into avenues to alleviate technical and user issues. Owing to connectivity problems, video visits are not always viable, especially for specific populations, such as those with limited digital access and literacy; this issue may worsen health inequity. A future focus for policy makers should consider inclusion of telephonic services as a form of reimbursable telemedicine. Permanent expansion of low-cost or free broadband internet for at-risk populations is also critical. For health care systems, it will be imperative to improve the ease of use of telemedicine as well as to provide support for patients to access such services.

### Limitations

Although this study provided insights into video visit failures, limitations exist. Data were collected between March 1 and December 31, 2020, at the height of the COVID-19 pandemic. As such, further studies should focus on longer periods of time. The population examined was limited to patients residing in the Midwest and receiving care at a single institution, although it was an institution covering academic and community settings as well as urban and rural patients; future studies should assess telemedicine failures throughout the United States. We included data for a subset of patients completing a patient experience survey; respondents were more likely to be older and less socioeconomically disadvantaged, and response bias may be present. When we removed these variables from the survey, the associations for the remaining variables were similar, suggesting that overall results were not biased. Different technology approaches may yield different results (eg, different modalities), although our approach incorporated both an EHR-integrated and stand-alone option for clinics to use. In addition, video visit failures were determined by EHR and visit coding data—the limitation of this method could underestimate the number of true issues experienced. In late September 2020, the technology used for video visits was upgraded and continued to progress over time. In addition, clinicians who had a failed video visit scheduled with the integrated solution had a second chance to conduct the visit through the nonintegrated strategy, and this practice would not be reclassified in the EHR; thus, the finding that the integrated solution was superior to the stand-alone solution may be misleading. This practice may explain some of the higher success rates with visits scheduled using the integrated platform, although issues such as network connectivity are unlikely to be materially better with another platform.

## Conclusions

This quality improvement study found that patient factors, including sociodemographic characteristics and age, were dominantly and systematically associated with the success or failure of a video visit. As policy makers debate expanding telehealth coverage and hospital systems focus on investments, the consideration of patient support, equity, and friction should be kept in the forefront to ensure that underserved patients are not left to fall further behind. Underserved patients may become disproportionately challenged by decreases or cutbacks surrounding insurance coverage or reimbursement for telephone-based services, threatening to worsen health care disparities. Coverage of telephonic services may improve accessibility and equity across the age and ability spectrum.^12,28,29^ A broader understanding of the variability, associated factors, and learning curves for telemedicine may help guide the next phase of optimization and refinement of telemedicine as a primary medium for health care.

## Notes

Supplement. **eTable.** Multicovariate Mixed Effects Adjusted Odds Ratios **eFigure 1.** Adjusted Learning Curves for Video Visit Success From Providers and Patients Using the Bernstein Polynomial Spline Coefficients From Analysis 1 **eFigure 2.** Adjusted Learning Curves for Video Visit Success From Providers and Patients Using the Bernstein Polynomial Spline Coefficients From Analysis 2
