# Supplementary material for: Analysis of Clinician and Patient Factors and Completion of Telemedicine Appointments Using Video
Source: JAMA Netw Open. 2021 Nov 4;4(11):e2132917. doi: 10.1001/jamanetworkopen.2021.32917 (PMC8569484; doi:10.1001/jamanetworkopen.2021.32917)
Supplement: Supplement. — eTable. Multicovariate Mixed Effects Adjusted Odds Ratios eFigure 1. Adjusted Learning Curves for Video Visit Success From Providers and Patients Using the Bernstein Polynomial Spline Coefficients From Analysis 1 eFigure 2. Adjusted Learning Curves for Video Visit Success From Providers and Patients Using the Bernstein Polynomial Spline Coefficients From Analysis 2 [file jamanetwopen-e2132917-s001.pdf]

Supplementary Online Content

Crotty BH, Hyun N, Polovneff A, et al. Analysis of clinician and patient factors and completion of telemedicine appointments using video. *JAMA Netw Open*. 2021;4(11):e2132917. doi:10.1001/jamanetworkopen.2021.32917

eTable. Multicovariate Mixed Effects Adjusted Odds Ratios

eFigure 1. Adjusted Learning Curves for Video Visit Success From Providers and Patients Using the Bernstein Polynomial Spline Coefficients From Analysis 1

eFigure 2. Adjusted Learning Curves for Video Visit Success From Providers and Patients Using the Bernstein Polynomial Spline Coefficients From Analysis 2

This supplementary material has been provided by the authors to give readers additional information about their work.

© 2021 Crotty BH et al. *JAMA Network Open*.

eTable. Multicovariate Mixed Effects Adjusted Odds Ratios (referred to as "Analysis 2" in this text, a subgroup analysis including patients with multiple visits)

Characteristic	OR1 (95% CI2)		p-value
Clinician type			0.15
DO/MD/DPM	—		
APNP/PA/PA-C	0.87 (0.72, 1.05)		
Provider comfort with technology			<0.001
Strongly agree	—		
Agree	0.67 (0.53, 0.85)		<0.001
Disagree	0.16 (0.09, 0.26)		<0.001
Neutral	0.20 (0.14, 0.27)		<0.001
Strongly disagree	0.12 (0.05, 0.30)		<0.001
Not available	0.29 (0.19, 0.45)		<0.001
Patient Age (years)			<0.001
18 - 40	—		
41 - 65	0.57 (0.53, 0.61)		<0.001
65 - 80	0.37 (0.34, 0.41)		<0.001
80+	0.40 (0.35, 0.46)		<0.001
Patient race			<0.001
White	—		
American Indian or Alaska Native	0.77 (0.52, 1.14)		0.2
Asian	1.10 (0.89, 1.32)		0.4
Black or African American	0.77 (0.71, 0.84)		<0.001
Native Hawaiian or Other Pacific Islander	0.99 (0.37, 2.62)		>0.9
other/unknown/null	0.95 (0.82, 1.11)		0.6
Device used for the visit			<0.001
Android/iPhone	—	—	
Laptop or computer	1.55 (1.16, 2.07)		0.003

© 2021 Crotty BH et al. *JAMA Network Open*.

Other smartphone	0.58 (0.43, 0.78)	<0.001
Tablet	1.70 (1.21, 2.38)	0.002
Not available	0.81 (0.53, 1.26)	0.4
Median income		0.01
$9500-$45,000	—	
$45,001-$75,000	1.13 (1.05, 1.22)	0.004
$75,001-$213,000	1.17 (1.06, 1.30)	0.002
Not available	1.12 (0.91, 1.39)	0.3
High Speed Internet*		0.87
High	—	
Medium	0.97 (0.90, 1.04)	0.4
Low	0.98 (0.89, 1.08)	0.7
NA	0.99 (0.80, 1.23)	>0.9
Visit platform		<0.001
Integrated platform	-	
Non-integrated platform	0.61 (0.57, 0.65)	
1OR = Odds Ratio, 2CI = Confidence Interval		

* Residential fixed high speed connections derived from Federal Communications Commission data. High denotes >800 connections capable of at least 10 Mbps download speeds and 1 Mbps upload / 1,000 households; medium 601-800 connections / 1,000 households, and low ≤600 connections / 1,000 households.

© 2021 Crotty BH et al. *JAMA Network Open*.

eFigure 1. Adjusted Learning Curves for Video Visit Success From Providers and Patients Using the Bernstein Polynomial Spline Coefficients From Analysis 1

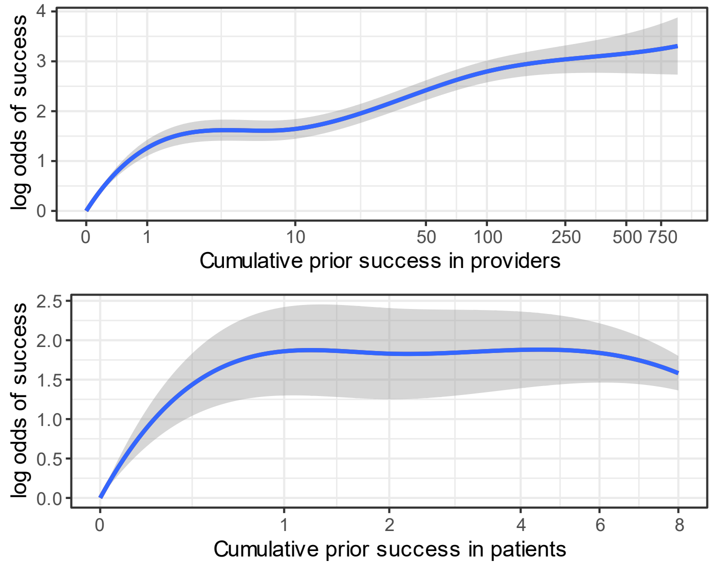

eFigure 2. Adjusted Learning Curves for Video Visit Success From Providers and Patients Using the Bernstein Polynomial Spline Coefficients From Analysis 2

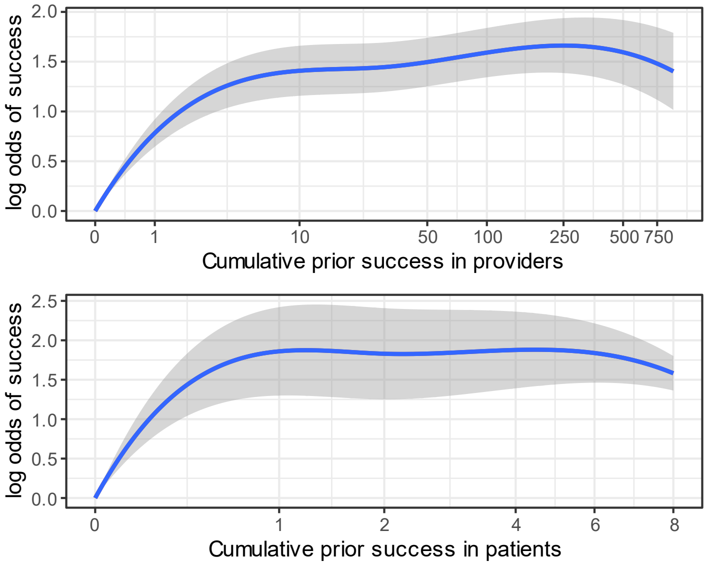

© 2021 Crotty BH et al. *JAMA Network Open*.
